# Microstructure Abnormalities of Diffusion Tensor Imaging Measures in First‐Episode, Treatment‐Naïve Adolescents With Major Depressive Disorder: An Integrated AFQ and TBSS Study

**DOI:** 10.1002/brb3.70416

**Published:** 2025-03-13

**Authors:** Wenjie Zhang, Chan Zhang, Jinyuan Zhao, Jiajing Cui, Jinji Bai, Xuan Deng, Junjun Ji, Ting Li, Yu Wang, Kefeng Li, Yunhui Qu, Junfeng Li

**Affiliations:** ^1^ Department of Radiology Heping Hospital Affiliated to Changzhi Medical College Changzhi Shanxi China; ^2^ Changzhi Key Lab of Functional Imaging for Brain Diseases Heping Hospital Affiliated to Changzhi Medical College Changzhi Shanxi China; ^3^ Department of Radiology Rizhao Hospital of Traditional Chinese Medicine Rizhao Shandong China; ^4^ Department of Psychiatry Changzhi Mental Health Center Changzhi Shanxi China; ^5^ Faculty of Applied Sciences Macao Polytechnic University Macao SAR China; ^6^ Department of Radiology, Yantai Yuhuangding Hospital Qingdao University Yantai Shandong China

**Keywords:** adolescents, automated fiber quantification, diffusion tensor imaging, major depressive disorder, tract‐based spatial statistics

## Abstract

**Purpose:**

Structural changes during depressive episodes in adolescents with major depressive disorder (MDD) remains unclear due to participant heterogeneity, illness chronicity, and medication confounders. This study aimed to explore white matter (WM) microstructural changes in first‐episode, treatment‐naïve adolescents with MDD using an integrated diffusion tensor imaging (DTI) approach.

**Method:**

We recruited 66 subjects, including 37 adolescents with MDD and 29 healthy controls. Two main DTI techniques, automated fiber quantification (AFQ) and tract‐based spatial statistics (TBSS), were used to analyze fractional anisotropy (FA), axial diffusivity (AD), radial diffusivity (RD), and mean diffusivity (MD) in WM tracts. DTI measures were then correlated with the depressive symptoms evaluated by Hamilton Depression Rating Scale scores (HAMD‐17).

**Findings:**

In AFQ, MDD patients showed significant segmental differences in WM tracts compared to controls, including a negative correlation between SLF AD values and depression severity. TBSS revealed reduced FA in the cingulum, forceps minor, inferior fronto‐occipital fasciculus, inferior longitudinal fasciculus, SLF, and uncinate fasciculus in MDD.

**Conclusion:**

Our integrated DTI analysis in a unique first‐episode, medication‐naïve cohort revealed microstructural changes in adolescent MDD not previously reported. These findings may provide imaging markers for early detection and enhance our understanding of depression pathology in youth.

## Graphical abstract

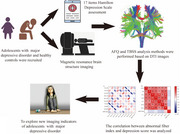

## 1 Introduction

Major depressive disorder (MDD), also known as clinical depression, is an emotional disorder that affects a wide range of people. Depression can make people feel like they are always unhappy, hopeless, and have lost interest in activities they used to enjoy (Alemu and Zeleke 2023). According to recent research results, the prevalence of depression among adolescents in the world reached 25% during the epidemic period in COVID‐19 (Racine et al. 2021). In order to achieve early detection and treatment of depression, many countries have proposed a surveillance program for depression during adolescence (Selph and McDonagh 2019). Adolescence is an important period for early evaluation and intervention treatment of depression, and timely measures to deal with depression can avoid its more serious consequences in the future.

Adolescence is an important transition stage in the process of human growth and development. Adolescents at this stage will experience great changes in psychology and physiology, and they need to make timely and appropriate adjustments to their self‐state. During this period, even the slightest changes in the environment may disrupt the balance and stability of the body's various systems, thereby increasing the possibility of depression in future life, and even leading to the existence of persistent subthreshold depression (Accrombessi et al. 2022). Therefore, abnormal maturational alterations in the brain may be linked to vulnerability to depression (Liang et al. 2023; Paus et al. 2008). Adolescents with MDD may develop long‐term social and psychological problems, leading to substance abuse, occupational dysfunction, and interpersonal disorders, and they are prone to relapse in adulthood (Rao and Chen 2009). According to current research, depression is caused by a breakdown in communication between brain regions that process and regulate emotions (Rai et al. 2022). These breakdowns may be caused by changes in the structural integrity of the pathways that typically permit efficient communication between these circuits (Lee et al. 2020). Therefore, understanding the neurobiological changes and underlying etiology of adolescent depression can help us to provide more substantial treatment programs, and even prevent the occurrence of adult depression and eliminate a series of health and social problems caused by depression.

Diffusion tensor imaging (DTI) is a magnetic resonance imaging (MRI) technique that can detect the orientation and integrity of white matter (WM) fiber tracts by monitoring the diffusion of water molecules in brain tissue (van Velzen et al. 2020). Using DTI, four different diffusion measures can be measured: fractional anisotropy (FA), mean diffusivity (MD), axial diffusivity (AD), and radial diffusivity (RD). Automatic fiber quantification (AFQ) is a tool for calculating WM fiber properties in whole‐brain tractography. The whole‐brain deterministic fiber tract imaging mainly constructs the brain fiber by detecting the main direction of the diffusion movement of water molecules in WM (Yeatman et al. 2012). Tract‐based spatial statistics (TBSS) is a widely used method in DTI analysis, which is a whole‐brain approach (Smith et al. 2006). This method addresses smoothness and misalignment problems in diffusion MRI‐based group analysis studies and offers specific processing procedures (Bach et al. 2014). A study of MDD in adolescents found that the FA value of the bilateral uncinate (UNC) tract was decreased, while the RD value was increased (LeWinn et al. 2014). Whole‐brain fiber analysis showed that treatment‐naïve adolescents with MDD had decreased FA values, increased RD and MD values, and unchanged AD values in the corpus callosum (CC) by TBSS analysis (Aghajani et al. 2014). In the study of F. Wu et al. (2019) on first‐episode adolescent depression, it was also found that the FA value of the left UNC in the depressed group was significantly lower than that in the healthy control (HC) group. In addition, a meta‐analysis of MDD in adolescents and adults revealed the decrease of FA values in corpus callosum, inferior frontal occipital tract, and corticospinal tract, and also showed that MDD would cause extensive damage and destruction of WM microstructure (Zhou et al. 2022). However, there are relatively few studies on first‐episode, treatment‐naïve adolescents with MDD using AFQ and TBSS methods.

In this study, we investigated the alterations in WM microstructure in adolescents with MDD using AFQ and TBSS analyses. While AFQ analysis is rarely used in studies on adolescents with MDD, TBSS analysis has been effectively employed in several investigations. To lessen the variation in results that could be caused by the different approaches, we employed two different analysis techniques (Deng et al. 2018). The primary goals of this study were to (1) compare the DTI measurements of adolescents with MDD with those of HCs, (2) investigate the relationship between the DTI measurements and depression severity, and (3) compare the findings of this investigation with those of other studies on adolescents with MDD.

## 2 Materials and Methods

### 2.1 Participants and Procedures

This study was conducted in accordance with the Declaration of Helsinki and approved by the Ethics Committee of Changzhi Medical College (IRB: CZMC‐20205). Informed consent was obtained from all participants. The questionnaire and MRI examination were performed in minors after obtaining the consent of their parents and themselves.

Participant recruitment: Between January 2020 and March 2023, a total of 37 adolescents with MDD were recruited from the Changzhi Mental Health Center. WeChat, advertising, and other methods were used to recruit 29 age‐ and sex‐matched HCs.

Inclusion criteria: (1) An assessment of the symptomatology, disease severity, and course of depression based on the *Diagnostic and Statistical Manual of Mental Disorders Fifth Edition* (DSM‐V); a Hamilton Rating Scale for Depression (HAMD‐17) scale score of ≥ 7 for patients suffering their first episode and who are treatment naïve; (2) In the HC group, there were no mental illnesses; (3) Both groups had no history of alcohol or drug abuse or other physical illnesses; (4) Based on the Family History‐Research Diagnostic Criteria (FH‐RDC) interview, first‐degree relatives have no history of mental illness; (5) Participants can volunteer and cooperate with MRI scanning; and (6) right‐handed participants are all present.

Exclusion criteria: (1) Other mental diseases coexisting with depression in MDD patients; (2) A history of substance abuse and alcoholism; (3) The HC group met any kind of mental illness in the DSM‐V or the HAMD‐17 score was ≥ 7; (4) Injuries or surgeries to the brain in the past; and (5) in pregnancy or during menstruation.

Clinical data such as age, gender, BMI, and medical history are collected by professionally trained doctors. In this study, depression was diagnosed according to DSM‐V criteria by two experienced clinical psychiatrists. HAMD‐17 scores were used to assess the severity of depression.

### 2.2 Image Acquisition and Preprocessing

3.0 T MAGNETOM Skyra Magnetic Resonance Instrument (Siemens, Germany) was equipped with a 32‐channel head coil for MRI imaging. T1WI, T2‐FLAIR, DTI, and 3D‐T1 sequences were used in both the adolescent MDD and HC groups. We instructed participants to keep their eyes closed and remain still, using a foam head cushion to fix their head position. All participants were required to wear noise‐reducing headphones and remain calm and awake during the MRI scan.

Parameters used in DTI were as follows: repetition time (TR) = 12,000 ms, echo time (TE) = 77 ms, slice thickness = 2 mm, no gap, field of view (FOV) = 224 × 224 mm, matrix size = 112 × 112, voxel resolution = 2 × 2 × 2 mm, diffusion direction = 30; *b* value = 1000. These parameters were used for the 3D‐T1 structure phase sequence: repetition time (TR) = 2530 ms, echo time (TE) = 2.98 ms, slice number = 192, slice thickness = 1 mm, field of view (FOV) = 256 × 256 mm, and voxel resolution = 1 × 1 × 1 mm.

FMRIB Software Library (FSL) version 6.0 (https://fsl.fmrib.ox.ac.uk/fsl) was used to preprocess DTI. We used the FSL “eddy” tool to estimate and correct eddy current‐induced distortions and gross participant movement (Andersson and Sotiropoulos 2016). Next, the Brain Extraction Tool (BET) was used to create individual brain masks with 0.20 as the threshold. Finally, FDT's DTIFIT tool was used to fit every voxel of the brain mask with a tensor model and to compute the voxel‐wise eigenvalues (λ1, λ2, and λ3) and eigenvectors, from which the diffusion measures FA, AD, RD, and MD were calculated.

### 2.3 AFQ Analysis

Participants' WM tracts in their brains were automatically extracted using automated fiber quantification software (AFQ, http://github.com/jyeatman/AFQ) (Yeatman et al. 2012). The AFQ process mainly includes five steps: (1) Using deterministic algorithms to track whole‐brain fibers. (2) Segmenting fibers using regions of interest (ROIs). (3) Using probability maps to refine fibers. (4) Cleaning fiber tracts using the outlier rejection algorithm. (5) On each fiber tract, 100 equidistant nodes were measured for diffusion metrics (FA, MD, AD, and RD).

As a result of the threshold setting utilized in fiber tracking (Hu et al. 2023), the AFQ approach does not detect all tracts in all subjects, so if a node only has a few fibers or a single fiber passes through a voxel, values are reported as missing. Patients with missing data were eliminated from the analysis of each fiber tract, and the degrees of freedom were modified appropriately. Based on the fiber tract treatment of each participant, 18 fiber bundles were selected for analysis. Table S1 provides the tract findings.

### 2.4 TBSS Analysis

We used both whole‐brain TBSS to examine WM microstructure. Voxel‐wise statistical analysis of the FA maps was conducted using the TBSS toolbox in FSL. First, FMRIB's non‐linear registration tool (FNIRT) was used to align individual FA images to the FMRIB58_FA standard‐space image. Next, a mean FA skeleton is generated and refined to create a mean FA skeleton, which represents the centers of all beams common to the whole group. For avoiding partial volume artifacts and excluding peripheral tracts, we set the mean skeleton threshold at an FA value of ≥ 0.35. In the final step, the aligned FA images of each participant were projected onto the mean FA skeleton. Similarly, MD, AD, and RD data were projected onto the skeleton using FA registration and skeleton projection parameters. A voxel‐wise permutation‐based analysis was then conducted using the skeletonized FA, MD, AD, and RD data. The analysis included age and gender as confounders. We performed nonparametric permutation‐based testing with FSL's randomize function. After 5000 permutations, the results were deemed significant at the *p* < 0.05 level according to permutation‐based nonparametric inference (Okazaki et al. 2020). Multiple comparisons were taken into account by threshold‐free cluster enhancement (TFCE) and family‐wise error rate correction (FWE).

### 2.5 Statistical Analyses

We used SPSS 26.0 to analyze general clinical data. Two‐sample independent *t*‐tests or Mann–Whitney *U* tests were used to compare continuous variables, including age, BMI, HAMD‐17 score, and diffusion measures in TBSS analysis. A chi‐square test was used to compare categorical data.

In AFQ analysis, for the detailed WM property analysis of each tract, mean values and standard errors of the means were calculated, along with point‐wise *t*‐tests performed for 100 randomly spaced nodes along the tract's length. FA, MD, AD, and RD profiles were computed at 100 equidistant nodes along the 18 tracts, respectively. To analyze the four outcomes (FA, MD, AD, and RD), we used a multiple‐comparison correction method. As covariables, age and gender were taken into account. The diffusion values extracted for significant nodes were correlated with HAMD‐17 scores using Pearson correlation analysis. A false discovery rate (FDR) procedure (*q* = 0.05) was implemented to correct for false positive inflation caused by multiple comparisons.

## 3 Results

### 3.1 Demographic and Clinical Characteristics

There were no significant differences in age, gender, and BMI between the MDD and HC groups. The MDD group had substantially greater HAMD‐17 scores (*p* < 0.001) than the HC group (Table 1).

**TABLE 1:** Clinical characteristics of the participants.

Subject characteristics	HC (*n* = 29)	MDD (*n* = 37)	*p* value
Age (years)	15.8 ± 2.2	15.2 ± 1.5	0.18
Gender (F/M)	18 F/11 M	28 F/9 M	0.23
BMI	21.5 ± 4.3	19.7 ± 3.5	0.06
HAMD‐17 scores	1.1 ± 1.5	18.6 ± 6.1	< 0.001

*Note*: The data are presented as the mean ± standard deviation (SD) or percentages. Group differences were assessed using either chi‐square analysis or independent *t*‐tests. A *p* value < 0.05 was considered to indicate statistical significance.

Abbreviations: BMI, body mass index; F, female; HAMD‐17, Hamilton Depression Scale (17 items); HC, healthy control; M, male; MDD, major depressive disorder.

### 3.2 Group Differences in Diffusion Measures Assessed by AFQ Analysis

There were no significant differences in the mean FA, MD, AD, or RD between the 18 WM tracts that were analyzed for each participant. However, we found segmental differences between the MDD and HC groups in several tracts (Figure S1).

**FIGURE 1:**
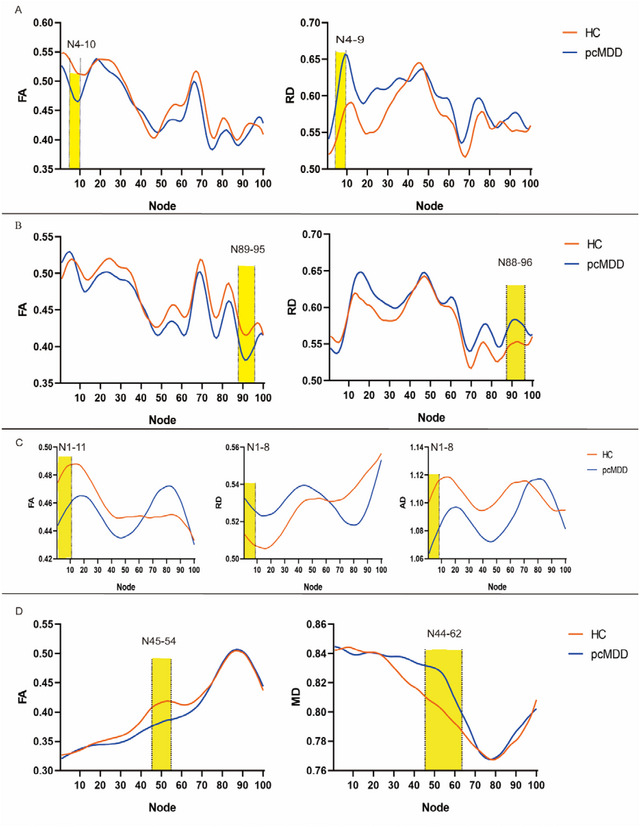
Automated fiber quantification (AFQ) reveals nodes in tracts with significant differences in diffusion measures between pubertal children with major depressive disorder (pcMDD) and healthy controls (HCs). In (A), nodes of the left IFO with a significant difference in FA (left panel) and RD (right panel) are shaded in yellow. In (B), nodes of the right IFO with a significant difference in FA (left panel) and RD (right panel) are shaded in yellow. In (C), nodes of the right SLF with a significant difference in FA (left panel), RD (middle panel) and AD (right panel) are shaded in yellow. In (D), nodes of the left UNC with a significant difference in FA (left panel) and MD (right panel) are shaded in yellow. The solid lines represent the mean FA, MD, RD, and AD values and are colored blue and orange for pcMDD and HCs, respectively. AD = axial diffusivity; FA = fractional anisotropy; IFO = inferior frontal‐occipital fasciculus; MD = mean diffusivity; RD = radial diffusivity; SLF = superior longitudinal fasciculus; UNC = uncinate fasciculus.

Figure 1 and Table 2 show the segmental differences of tracts between the groups. For the left inferior fronto‐occipital fasciculus (IFO), compared with the HC group, the FA value between Nodes 4 and 10 was decreased in the MDD group, while the RD value between Nodes 4 and 9 was increased. The representation of the range of abnormal nodes on the fiber tract was shown in Figure 1A. A significantly decreased FA was observed between Nodes 89 and 95 of the right IFO, while a significantly increased RD was observed between Nodes 88 and 96 in MDD compared to HC group. Figure 1B shows these anomalies mapped to the tract. For the right superior longitudinal fasciculus (SLF), significantly decreased FA was found between Nodes 1 and 11, significantly decreased AD was found between Nodes 1 and 8, and significantly increased RD was found between Nodes 1 and 8 in MDD relative to HC group. The anomalies were shown mapped onto a representation of the tract in Figure 1C. For the left UNC tract, significantly decreased FA was found between Nodes 45 and 54, and significantly increased MD was found between Nodes 44 and 62 in MDD relative to HC group. These abnormal nodes were mapped to the tract in Figure 1D.

**TABLE 2:** Nodes with differences in diffusion measures between pubertal children with major depressive disorder (MDD) and healthy controls (HCs).

Diffusion measures	Fiber tract	Abnormal node range
Fractional anisotropy	Left inferior fronto‐occipital fasciculus	4–10
Fractional anisotropy	Right inferior fronto‐occipital fasciculus	89–95
Fractional anisotropy	Right superior longitudinal fasciculus	1–11
Fractional anisotropy	Left uncinate	45–54
Mean diffusivity	Left uncinate	44–62
Axial diffusivity	Right superior longitudinal fasciculus	1–8
Radial diffusivity	Left inferior fronto‐occipital fasciculus	4–9
Radial diffusivity	Right inferior fronto‐occipital fasciculus	88–96
Radial diffusivity	Right superior longitudinal fasciculus	1–8

### 3.3 Group Differences in Diffusion Measures According to TBSS Analysis

**FIGURE 2:**
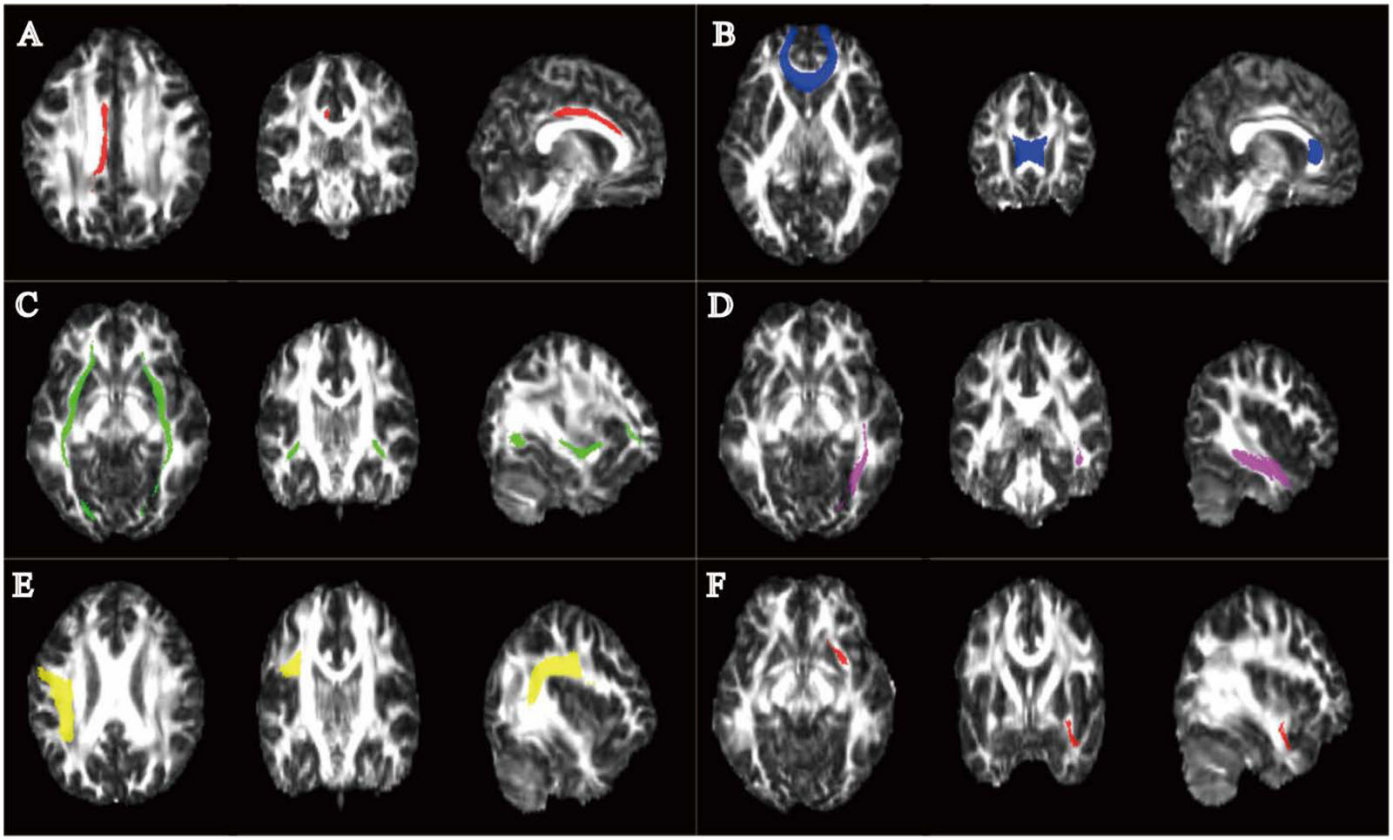
Tract‐based spatial statistics (TBSS) results comparing fractional anisotropy (FA) values in adolescents with major depressive disorder (MDD) and healthy controls (HCs). Note: The FA maps show axial, coronal, and sagittal views (from left to right). Red voxels represent the mean white matter skeleton from the entire sample, and other colored voxels represent white matter regions with lower FA in pubertal children with major depressive disorder (pcMDD) compared with HCs (*p* < 0.05, threshold‐free cluster enhancement [TFCE]‐corrected). (A) Right cingulum (cingulate gyrus, CinG). (B) Callosum forceps minor (CFM). (C) Bilateral inferior fronto‐occipital fasciculus (IFO). (D) Left inferior longitudinal fasciculus (ILF). (E) Right superior longitudinal fasciculus (SLF). (F) Left uncinate fasciculus (UNC).

We discovered some noteworthy differences between MDD and HC group (adjusted for TFCE). In all regions where there was a significant group difference, adolescents with MDD had reduced FA values compared with HC group (Table S2). We found significant differences in the right cingulum (cingulate gyrus [CinG]) (Figure 2A), callosum forceps minor (Figure 2B), bilateral IFO (Figure 2C), left ILF (Figure 2D), right SLF (Figure 2E), and left UNC fasciculus (Figure 2F).

### 3.4 Correlation Analysis Results

**FIGURE 3:**
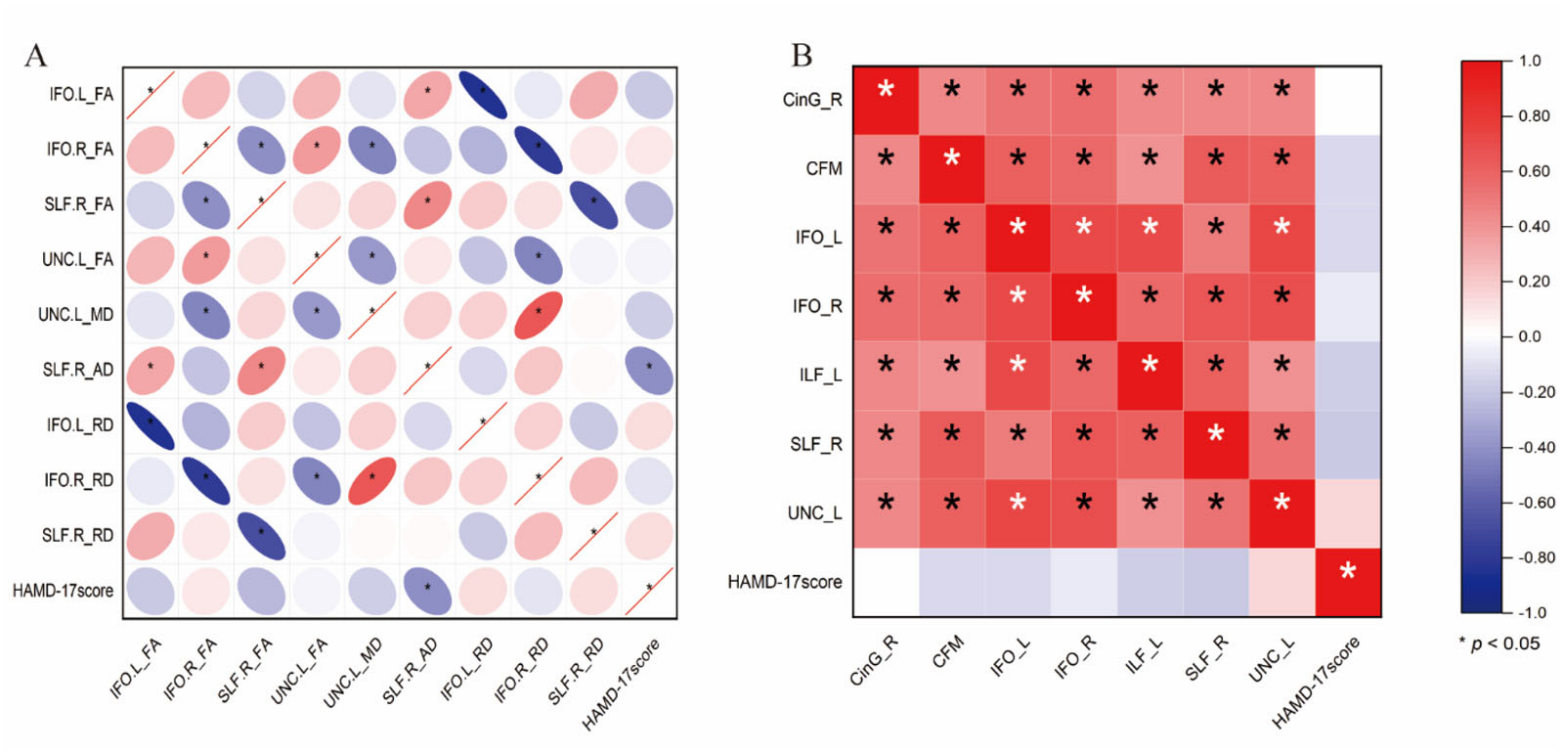
Correlations among fractional anisotropy (FA) values in white matter (WM) regions, mean values of nodes with differences, and HAMD‐17 scores. (A): Correlations between the mean diffusion measures of nodes with differences and HAMD‐17 score. (B): Correlations between the FA values of WM regions and HAMD‐17 scores. Spearman correlation was employed, with the correlation coefficient *r* values denoted using the color bar; * indicates statistical significance. AD: axial diffusivity; CFM: callosum forceps minor; CinG: cingulum (cingulate gyrus); FA: fractional anisotropy; HAMD‐17: Hamilton Depression Rating Scale; IFO: inferior fronto‐occipital fasciculus; ILF: inferior longitudinal fasciculus; L: left; MD: mean diffusivity; R: right; RD: radial diffusivity; SLF: superior longitudinal fasciculus; UNC: uncinate fasciculus.

The correlations between the mean diffusion measures of the different nodes and the HAMD‐17 score determined by AFQ analysis are illustrated in Figure 3A. For the right SLF, the mean AD at Nodes 1–8 was negatively correlated with the HAMD‐17 score in MDD (*r* = −0.37). No significant correlations were found between the other diffusion measures and HAMD‐17 scores according to the AFQ analysis. Moreover, there was no significant correlation between the FA value and HAMD‐17 score according to the TBSS analysis (Figure 3B).

## 4 Discussion

In this study, we found abnormal diffusion properties of WM fiber tracts in first‐episode, drug‐naïve adolescents with MDD compared with HCs by using DTI. AFQ analysis of DTI MRI scans revealed segmental anomalies between the bilateral IFO, right SLF, and left UNC fasciculus fiber tracts but no significant variations in the mean FA, MD, AD, or RD. We also found that, compared to HC group, MDD had lower FA values in the right cingulum, callosum forceps minor, bilateral IFO, left ILF, right SLF, and left UNC according to TBSS.

The limbic system's central component, the cingulum tract is a crucial component of Papageorge's circuit for emotion (Papez 1995). The parahippocampal region is connected to the cingulum bundles, which are the main intrahemispheric association pathways that link the splenium of the corpus callosum, the anterior cingulate cortex (ACC), and the posterior cingulate cortex (Long et al. 2023). Our tracking result for the cingulum in the TBSS analysis was around the cingulate gyrus. Corpus callosum forceps are mainly composed of fiber tracts in bilateral frontal lobes, which are closely related to cognitive and emotional processing (de Diego‐Adeliño et al. 2014; Paul et al. 2007). According to a meta‐analysis, the FA in the corpus callosum was lower in adolescents and young adults with MDD than in HC group according to TBSS (Zhou et al. 2022). An additional study revealed the correlation between the decrease of FA value in cingulate tract and the aggravation of depressive symptoms in MDD adolescents (Aghajani et al. 2014; Korgaonkar et al. 2011). Although there is no correlation between these two parameters in our study, we believe that the abnormality of cingulate bundles may disturb the information exchange between specific brain regions, which may lead to the disorder of memory, emotional control, and cognitive function. Therefore, we speculate that the abnormality of cingulate tract may play an important role in the pathophysiology of adolescent depression.

The lateral boundary of the caudate nucleus is where the IFO extends backward from the frontal lobe to the posterior, temporal, and occipital lobes. In particular, the ventral attention system offers anatomical connections for spatial attention (Schmahmann et al. 2007). The IFO is an important part of frontal‐subcortical circuits and plays an important role in it (Na et al. 2016; Wei et al. 2020). According to a systematic review, the abnormality of frontal‐subcortical circuits is closely related to the occurrence of depression (Sexton et al. 2009). Previous studies on suicidal depression patients found that the FA value of IFO in depression group was significantly lower than that in HC group (Zhang et al. 2022). In the AFQ analysis, we also found a segmental difference between the MDD group and the HC group in which the FA value was decreased and the RD value was increased. Compared with HC group, the decrease of FA in MDD group may reflect the extensive decrease of WM structural integrity, regional myelination level, axon density, and diameter, while the increase of RD may indicate the existence of potential changes in myelination integrity, such as demyelination or myelination disorder. Changes in the RD in the IFO may have an impact on cognitive suppression of sensory stimuli and emotions as well as sensory integration (Lai and Wu 2013). Therefore, we hypothesize that structural abnormalities in the IFO may serve as an imaging marker of suicidal tendencies in adolescents with MDD. However, this correlation needs to be further studied and analyzed.

SLF is the main fiber connecting temporal lobe, occipital lobe, parietal lobe, and frontal lobe, which plays an important role in processing visual information, distinguishing things, and regulating emotions (Haghshomar et al. 2018). In our study, we found that the FA values of MDD were lower than those of HC group in the ILF and SLF. In previous studies of MDD, the destruction of these tracts has not been reported, although it has been implicated in both adult depression and Parkinson's disease (Qiu and Li 2018; Shen et al. 2017; J. Wu et al. 2018). Compared to HC group, the AFQ analysis also revealed a decrease in segmental FA and AD values and an increase in RD values in the MDD group. AD is closely associated with fiber diameter or organization: low values may reflect axonal loss or fragmentation, especially for MDD patients, and may be a good biomarker for this disease (Vai et al. 2020) In addition, the correlation analysis revealed a negative correlation between the segmental AD value in the SLF and depression severity. Therefore, we speculate that the ILF and SLF may represent new imaging markers for the diagnosis of adolescents with MDD, and correlation analysis further supports the feasibility of the SLF as a marker.

Furthermore, our TBSS and AFQ analyses revealed decreased FA values in the left UNC in adolescents with MDD. UNC plays a crucial role in the cortico‐limbic system by connecting limbic system regions such as the amygdala with the medial orbital prefrontal cortex (OMPFC) and the ACC. The ventral cortical marginal loop, which is composed of amygdala, OMPFC, and ACC, is considered to be related to emotional stimulation (Connolly et al. 2013; Ho et al. 2013). Previous studies on adolescent depression also showed that FA value decreased in UNC, which was consistent with the findings of this study (Cullen et al. 2010; LeWinn et al. 2014). It is worth noting that the decrease of FA value was accompanied by the increase of MD value and the unchanged AD value, which may indicate that demyelination (increased MD value) without axonal loss (unchanged AD value) may be the basis for the decrease of FA value in UNC segments (Lebel and Deoni 2018). The destruction of the UNC structure has also been mentioned in some reports of adult depression (de Kwaasteniet et al. 2013; Dillon et al. 2018; Sacchet et al. 2014). Based on previous literature reports and the results of this study, we suggest that the decreased FA value of UNC may be an important imaging basis for the onset of depression, especially after the inclusion of early onset untreated population studies.

In our study, there are some limitations. First, due to our study's cross‐sectional design, we cannot infer causal relationships between underlying WM microstructure and depression among adolescents. In addition, a deterministic tractography method was used for analyzing the DTI data, which may have limited the ability to display fiber crossings and fiber kisses. A third limitation is that we did not distinguish between participants who were in remission or those in whom the disease was active, which may have influenced our results.

## 5 Conclusion

Our AFQ and TBSS methods detected mild changes in WM, allowing us to identify abnormalities in fiber tracts with a greater degree of spatial precision. In addition, for the first time, we discovered fiber tract changes in the ILF and SLF in first‐episode, drug‐naïve adolescents with MDD. These findings may lead to the identification of new imaging markers for detecting adolescents with depression in the future and may contribute to a greater understanding of its pathology.

## Author Contributions

**Wenjie Zhang**: formal analysis, investigation, methodology, writing–original draft, writing–review and editing. **Chan Zhang**: formal analysis, investigation, methodology, writing–original draft, writing–review and editing. **Jinyuan Zhao**: investigation, methodology, writing–review and editing. **Jiajing Cui**: data curation, investigation, writing–review and editing. **Jinji Bai**: formal analysis, investigation, writing–review and editing. **Xuan Deng**: investigation, writing–review and editing. **Junjun Ji**: data curation, methodology, resources, supervision, writing–review and editing. **Ting Li**: conceptualization, resources, software, supervision, writing–review and editing. **Yu Wang**: conceptualization, resources, software, writing–review and editing. **Kefeng Li**: conceptualization, data curation, supervision, writing–review and editing. **Yunhui Qu**: conceptualization, methodology, supervision, writing–original draft, writing–review and editing. **Junfeng Li**: conceptualization, funding acquisition, project administration, resources, supervision, writing–review and editing.

### Peer Review

The peer review history for this article is available at https://publons.com/publon/10.1002/brb3.70416

## Supporting information



Supporting Information

## Data Availability

The data that supports the findings of this study are available from the corresponding authors upon reasonable request.
