# Supplementary material for: Microstructure Abnormalities of Diffusion Tensor Imaging Measures in First‐Episode, Treatment‐Naïve Adolescents With Major Depressive Disorder: An Integrated AFQ and TBSS Study
Source: Brain Behav. 2025 Mar 13;15(3):e70416. doi: 10.1002/brb3.70416 (PMC11905106; doi:10.1002/brb3.70416)
Supplement: Supplementary file 1 — Supporting Information [file BRB3-15-e70416-s001.docx]

Supplemental materials
 
Figure S1. Automated fiber quantification (AFQ) reveals significant tracts with node differences in diffusion measures between adolescents with major depressive disorder(MDD) and healthy controls (HCs). (A) Left inferior fronto-occipital fasciculus; (B) Right inferior fronto-occipital fasciculus; (C) Right superior longitudinal fasciculus; (D) Left uncinate fasciculus.
 
 
 
Table S1 Successful identification rate for each of 18 fiber tracts in the first-episode, treatment-naïve adolescents with major depressive disorder(MDD) and healthy controls (HCs) by AFQ.
Index	Tract	Total subjects (N0)	No. of subjects showing successful tract identification (N1)	Ratio (N1/N0)
1	Left ATR	66	66	1
2	Right ATR	66	66	1
3	Left Corticospinal	66	66	1
4	Right Corticospinal	66	66	1
5	Left Cingulum	66	66	1
6	Right Cingulum	66	66	1
7	Callosum Forceps Major	66	65	0.98
8	Callosum Forceps Minor	66	66	1
9	Left IFO	66	66	1
10	Right IFO	66	66	1
11	Left ILF	66	65	0.98
12	Right ILF	66	66	1
13	Left SLF	66	66	1
14	Right SLF	66	66	1
15	Left Uncinate	66	66	1
16	Right Uncinate	66	66	1
17	Left Arcuate	66	64	0.97
18	Right Arcuate	66	63	0.95

 
 
 
Table S2 White matter (WM) regions with significant differences between adolescents with major depressive disorder(MDD) and healthy controls (HCs) by TBSS.
Diffusion measures	Regions	MDD	HC	t value	P value
FA	CinG.R	0.55±0.03	0.57±0.03	2.14	0.036
FA	CFM	0.56±0.02	0.57±0.02	2.88	0.005
FA	IFO.L	0.52±0.02	0.53±0.02	2.26	0.027
FA	IFO.R	0.52±0.02	0.53±0.02	2.05	0.044
FA	ILF.L	0.49±0.02	0.50±0.02	2.07	0.043
FA	SLF.R	0.49±0.02	0.51±0.02	2.40	0.019
FA	UNC.L	0.49±0.04	0.51±0.04	2.09	0.041

Abbreviations: Right (R); Left (left); cingulum(cingulate gyrus, CinG); callosum forceps minor (CFM); inferior fronto-occipital fasciculus (IFO); inferior longitudinal fasciculus(ILF); superior longitudinal fasciculus (SLF); uncinate fasciculus (UNC); fractional anisotropy (FA); Tract-based spatial statistics (TBSS); adolescents with major depressive disorder (MDD). Data are presented as mean ± standard deviation (SD) or as percentages. Group differences were assessed using either Chi-square analysis or Independent t-tests. A P-value < 0.05 was considered statistically significant.
